# Prognostic value of pre-procedural left ventricular strain for clinical events after transcatheter aortic valve implantation

**DOI:** 10.1371/journal.pone.0205190

**Published:** 2018-10-11

**Authors:** Noriko Suzuki-Eguchi, Mitsushige Murata, Yuji Itabashi, Kousuke Shirakawa, Memori Fukuda, Jin Endo, Hikaru Tsuruta, Takahide Arai, Kentaro Hayashida, Hideyuki Shimizu, Keiichi Fukuda

**Affiliations:** 1 Department of Cardiology, School of Medicine, Keio University, Tokyo, Japan; 2 Center for Preventive Medicine, Keio University Hospital, Tokyo, Japan; 3 Department of Laboratory Medicine, School of Medicine, Keio University, Tokyo, Japan; 4 Cardiovascular Surgery, School of Medicine, Keio University, Tokyo, Japan; University of Bern, University Hospital Bern, SWITZERLAND

## Abstract

**Background:**

Transcatheter aortic valve implantation (TAVI) is an alternative therapy for surgically high-risk patients with severe aortic stenosis (AS). Although TAVI improves survival of patients with severe AS, the mechanism of this effect remains to be clarified. We investigated the effects of TAVI on left ventricular (LV) function and identified the predictive parameters for cardiac events after TAVI.

**Methods and results:**

We studied 128 patients with severe symptomatic AS who underwent TAVI. Echocardiographic assessments were performed before and after TAVI. In addition to the conventional echocardiographic parameters such as LV ejection fraction (LVEF) and LV mass index (LVMI), the LV global longitudinal strain (GLS) and early diastolic peak strain rate (SR_E) using two-dimensional speckle tracking echocardiography were also evaluated. All patients were assessed for clinical events including major adverse cardiac events and stroke according to Valve Academic Research Consortium-2 criteria. GLS, early diastolic peak velocity (eʹ), aortic regurgitation (AR) severity, and SR_E were significantly improved after TAVI. Thirteen patients had an event during the observational period of 591 days (median). Patients with events had higher LVMI, more severe AR, and worse GLS compared to those without events. Furthermore, receiver-operating curve analysis revealed that GLS was the strongest predictor for clinical events (p = 0.009; area under the curve, 0.73).

**Conclusion:**

Preoperative LV geometric deformation and dysfunction, as a consequence of the cumulative burden of pressure overload, improved after TAVI and could predict cardiac events after TAVI.

## Introduction

Aortic stenosis (AS) is a common valvular heart disease, and conventional surgical aortic valve replacement (SAVR) is the therapy of choice for the majority of patients. However, for patients considered to be at surgically high risk, transcatheter aortic valve implantation (TAVI) has emerged as a less invasive option than surgical valve replacement over the past decade. Several recent studies have shown the feasibility and safety of TAVI during short-term and mid-term follow-up periods [1–3]. Lefevre et al. reported that all-cause 30-day mortality was lower in recent years and that there were significantly less major vascular complications, life-threatening bleedings, and major bleedings after TAVI compared to when TAVI was first introduced [3]. The early results are encouraging, with reported 30-day mortality rates less than 10% and 1-year survival rates more than 70% [4–9].

Several studies have demonstrated an improvement in left ventricular (LV) systolic function assessed by LV ejection fraction (LVEF), tissue Doppler, and speckle tracking strain imaging in patients with severe AS after SAVR during mid-term and long-term follow-up [10–12]. Recent studies also demonstrated recovery of LVEF and global longitudinal strain (GLS) after TAVI [13]. Furthermore, another study reported that TAVI induces faster recovery of LV geometry and greater reduction of the estimated LV filling pressure than traditional SAVR [14].

Recently, the ratio of global diastolic strain rate (SR) to mitral early diastolic velocity (E) (E/SR) was reported as a relatively novel parameter of LV relaxation and filling pressure [15]. Dahl et al. reported that the ratio of pre-operative E to early diastolic peak strain rate (SR_E; E/SR_E) was significantly associated with long-term post-operative survival and was superior to the E/early diastolic peak velocity (eʹ) ratio (E/eʹ) for patients with severe AS undergoing SAVR [16].

The aim of this study was to investigate the effect of TAVI on LV function and to identify echocardiographic predictors of clinical events after TAVI.

## Methods

### Study population

From October 2013 to July 2016, we retrospectively studied patients who underwent transthoracic echocardiography before and after (within 7 days) TAVI using Vivid-E9 or Vivid-7 ultrasound machines (GE Healthcare). One hundred twenty-eight patients were included in the main outcome analysis; 18 were excluded due to clinical trial (n = 17) or unanalyzable poor trace (n = 1). Table 1 presents the clinical characteristics of the study subjects. The institutional Review Board of Keio University approved this retrospective, observational cohort study and the requirement to obtain informed consent was waived (IRB No. 20160249). The study was conducted in accordance with the guidelines of the Declaration of Helsinki. The de-identified data request should be sent to the corresponding author (Murata M. muratam@keio.jp).

**Table 1:** Patient background.

Subject	128
Age (year)	83.7±4.23
Female (%)	84 (65.6)
BSA (m^2^)	1.43±0.201
HR (bpm)	69.8±11.7
Systolic BP (mmHg)	125±21.0
Diastolic BP (mmHg)	63.5±11.9
Comorbidity	
CAD (%)	44 (34.4)
HT (%)	93 (72.7)
CKD (%)	74 (57.8)
DM (%)	35 (27.3)
Medication	
Ca blocker (%)	68 (53.1)
ACE-I (%)	10 (7.81)
ARB (%)	51 (39.8)
β blocker (%)	33 (25.8)
Statin (%)	60 (46.9)
Diuretics (%)	69 (53.9)
Bioprosthetic valve	
Sapien XT (%)	118 (92.2)
Sapien 3 (%)	4 (3.13)
CoreValve (%)	6 (4.69)
BNP (pg/ml)	369±428

BSA, body mass index; HR, heart rate; BP, blood pressure; CAD, coronary artery disease; HT, hypertension; CKD, chronic kidney disease; DM, diabetes mellitis; ACE-I, angiotensin-converting enzyme inhibitor; ARB, angiotensin II receptor blocker; BNP, brain natriuretic peptide

### Echocardiography

All patients underwent standard echocardiography using a Vivid-7 or Vivid-E9 ultrasound system (GE Healthcare). Offline analyses were performed (EchoPAC; GE Healthcare) for all measurements. LV dimensions were obtained in the parasternal long axis with the M-mode cursor positioned just beyond the mitral leaflet tips, perpendicular to the long axis of the ventricle. LVEF was obtained using modified Simpson methods. LV mass index (LVMI) was calculated using the liner method (Cube formula). The mean transvalvular gradient was calculated using the Bernoulli formula. The aortic valve area was measured using the continuity equation. Early diastolic peak velocity (Eʹ) and late diastolic peak velocity (Aʹ) from Doppler tissue imaging were measured at the septal and lateral mitral annulus.

### Speckle tracking echocardiography

LV myocardial longitudinal function was evaluated using two-dimensional speckle tracking echocardiography (2DSTE). Offline analysis was performed using semi-automated 2D strain software (EchoPAC). First, the endocardial border was manually traced and the myocardial motion was tracked. The longitudinal strain and strain rate were measured in the apical long axis, 4-chamber, and 2-chamber views.

Wang et al. demonstrated that the strain rate during isovolumetric relaxation (SR_IVR) and during early LV filling (SR_E) had strong correlations with the time constant of LV pressure decay, and that E/SR_IVR and E/SR_E were more accurate for estimating LV filling pressure than E/eʹ [15]. Therefore, we also measured SR_E using 2DSTE.

The absolute value of the peak GLS was measured and the peak longitudinal strain rate during early diastole (SR_E) was calculated as an average of those measured in three views. Only the absolute values are referred to this study in all comparisons of GLS between groups according to the American Society of Echocardiography [17].

Twenty randomly selected patients were tested to determine the reproducibility of 2DSTE. The interclass correlation coefficients for interobserver and intraobserver reproducibility for 2DSTE were 0.93 (95% confidence interval [CI], 0.87–0.97), and 0.95 (95% CI, 0.93–0.97), respectively.

### Definitions of clinical outcomes

Clinical events were pre-specified as the primary end points of death and hospitalization due to congestive heart failure and stroke according to the Valve Academic Research Consortium (VARC)-2 criteria. The follow-up period was 591 days (median).

### Statistical analysis

Data are presented as mean value ± standard deviation (SD). Differences between groups were determined using the Student paired *t* test. Chi-square or Fisher exact tests were used to analyze the categorical data. We calculated the cumulative clinical events using the Kaplan-Meier method and compared the two curves using a log-rank test.

In addition, the area under the curve (AUC) of the model was calculated using the generalized U statistic; p<0.05 was considered statistically significant.

From the available information on echocardiographic parameters before TAVI, the following variables were of clinical interest in their relationship with clinical events: left ventricular diastolic dimension (LVDd), left ventricular systolic dimension (LVDs), Interventricular septum (IVS), posterior wall (PW), LVEF, LVMI, E, A, E/A, Dct, averaged e’, E/e’, aortic valve peak velocity, aortic valve mean pressure gradient, aortic valve area (AVA), GLS, SR_E and E/SR_E. These relationships were assessed through univariate logistic regressions of events. All statistical analyses were performed using JMP 11.0 software (SAS Institute, Cary, NC, USA).

## Results

Baseline characteristics of the study population are presented in Table 1. The mean age of the study population was 84±4.2 years and 65.6% of patients were female. Trans-femoral (TF)-TAVI was used in 113 cases, trans-apical (TA)-TAVI in 14 cases and direct-aortic (DA)-TAVI in 1 case.

Baseline echocardiographic parameters and measurements obtained within 7 days after TAVI are presented in Table 2.

**Table 2:** Echocardiographic data before and after TAVI.

Parameters	Before TAVI	After TAVI	P value
LVDd (mm)	44±5.7	44±5.3	0.77
LVDs (mm)	28±6.7	27±6.2	0.056
IVS (mm)	11±2.0	11±1.9	0.081
PW (mm)	11±1.5	10±1.4	0.025
LVEF (%)	62±13	64±11	0.063
LVMI (g/m^2^)	119±32	116±29	0.11
E (cm/s)	79±25	93±28	<0.0001
A (cm/s)	106±28	115±36	<0.0001
E/A	0.79±0.42	0.88±0.48	0.0001
Dct (ms)	268±93	251±88	<0.0001
Septal e' (cm/s)	3.9±1.2	4.2±1.3	<0.0001
Lateral e' (cm/s)	5.6±1.9	5.9±2.1	0.0018
Averaged e' (cm/s)	4.7±1.4	5.2±1.7	<0.0001
E/e'	17±7.7	20±8.6	0.015
Peak AV velocity (m/s)	4.5±0.7	2.2±0.4	<0.0001
AV mean PG (mmHg)	50±18	10±3.9	0.0003
AVA (cm^2^)	0.65±0.18	1.7±0.43	0.0008
AR (≧3) (%)	16 (13)	3 (2.3)	0.0025
MR (≧3) (%)	11 (8.6)	10 (7.8)	0.74
TR (≧3) (%)	7 (5.5)	8 (6.3)	0.66
GLS (%)	-15±4.4	-16±4.3	<0.0001
SR_E (/s)	0.78±0.34	0.90±0.37	<0.0001
E/SR_E (cm)	121±67	127±79	<0.0001

LVDd, left ventricular diastolic dimension; LVDs, left ventricular systolic dimension

IVS, Interventricular septum; PW, posterior wall; LVEF, left ventricular ejection fraction; LVMI, left ventricular mass index; Dct, deceleration time; AV, aortic valve; mean PG, mean pressure gradient; AVA, aortic valve area; AR, aortic regurgitation; MR, Mitral regurgitation; TR, tricuspid regurgitation; GLS, global longitudinal strain; SR_E, early diastolic peak strain rate.

At baseline, LV mass was increased but the LV wall thicknesses of the interventricular septum and posterior wall were within normal range. The mean preprocedural LVEF was 62±13% and peak longitudinal GLS was -15±4.4%, indicating that LV longitudinal systolic function was impaired. Low eʹ and high E/eʹ indicated impaired LV relaxation and increased LV filling pressure before TAVI, respectively.

Acute procedural success was achieved for all patients (100%). No patients required urgent cardiac surgery to manage complications.

### Effects of TAVI on echocardiographic parameters

Table 2 also shows that the peak AV velocity (4.5±0.7 m/s vs. 2.2±0.4 m/s; p<0.0001) and mean pressure gradient (50±18 mmHg vs. 10±3.9 mmHg; p = 0.0003) significantly decreased after TAVI. The parameters of LV relaxation including average eʹ (4.7±1.4 cm/s vs. 5.2±1.7 cm/s; p<0.0001) and SR_E (0.78±0.34 /sec vs. 0.90±0.37 /sec; p<0.0001) also significantly improved after TAVI, in keeping with improved LV relaxation after TAVI. Furthermore, GLS was significantly improved (-15±4.4% vs. -16±4.3%; p<0.0001) after TAVI, while there was no significant change in LVEF before and after TAVI (62±13% vs. 64±11%; p = 0.063). In contrast, increased E/SR_E was unexpectedly observed after TAVI (121±67 cm vs. 127±79 cm; p<0.0001).

### Clinical events after TAVI

**Fig 1:**
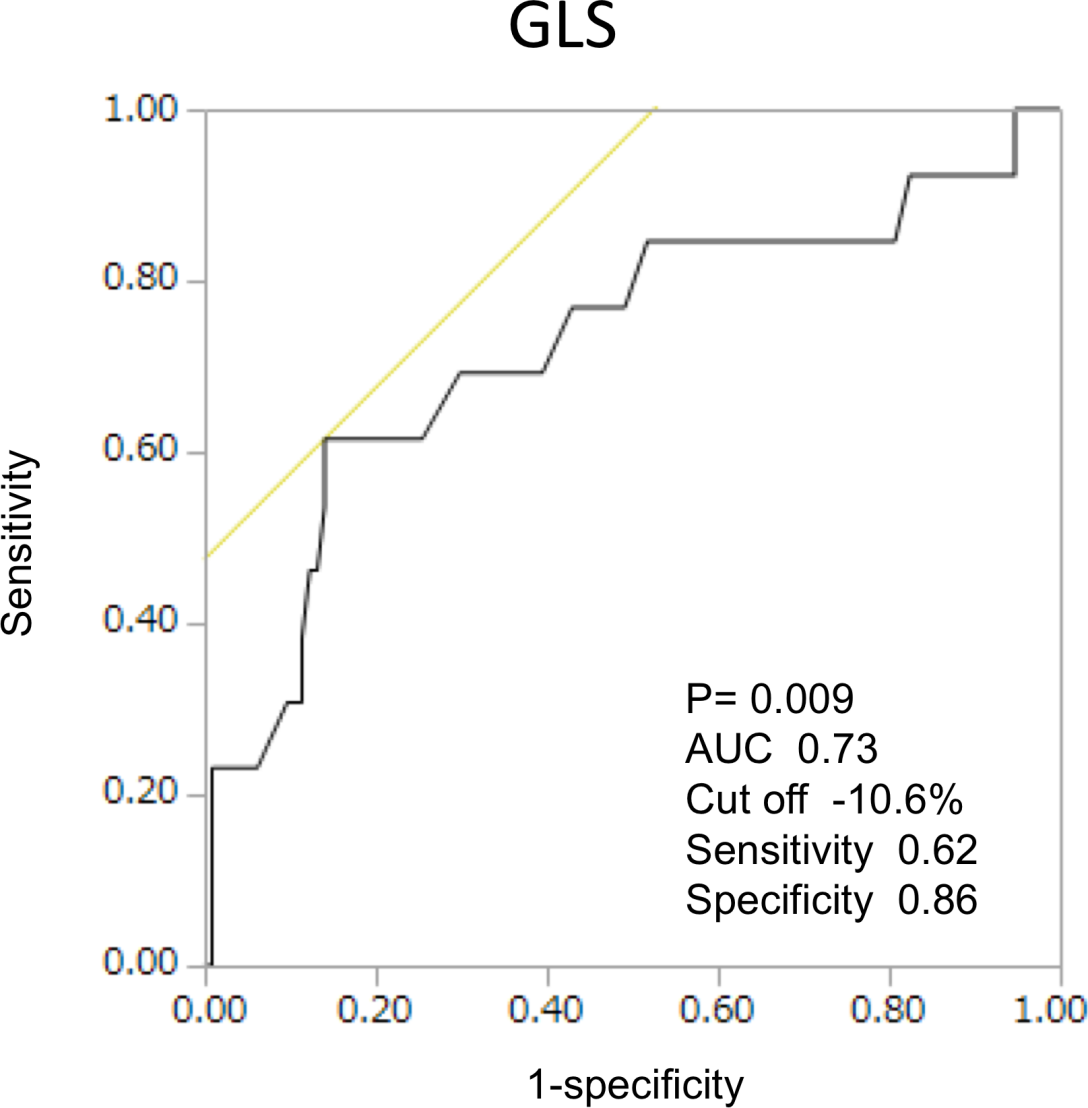
Receiver-operating characteristic (ROC) curves showing LV global longitudinal strain (GLS) for predicting events after transcatheter aortic valve implantation. AUC, area under the curve.

Thirteen patients had clinical events (2 cardiac deaths, 6 admissions due to heart failure, 5 strokes) during the observational period of 591 days (median). Table 3 shows the clinical and echocardiographic characteristics before TAVI in patients with and without clinical events. Patients with clinical events had worse GLS (-11.6±1.2% vs. -15.1±0.4%; p = 0.0061) compared to those without clinical events. During univariate regression analyses, only GLS (odds ratio [OR], 1.23; 95% CI, 1.05–1.45; p = 0.004) was associated with MACE after TAVI (Table 4). Receiver-operating characteristic (ROC) curve analysis identified GLS of -10.6% (AUC, 0.73) as the best cut-off value for predicting clinical events after TAVI (Fig 1).

**Table 3:** Clinical and echocardiographic characteristics in patients with and without events.

Parameters	Event (+)	Event (-)	P value
n	13	115	
Age (year)	84.2±1.19	83.6±0.39	0.6202
Female (%)	11 (84.6)	73 (63.5)	0.2166
CAD (%)	7 (53.9)	37 (32.2)	0.1336
HT (%)	10 (76.9)	83 (72.2)	1.000
CKD (%)	9 (69.2)	65 (56.5)	0.5553
DM (%)	4 (30.8)	31 (27.0)	0.7502
BNP (pg/ml)	462±119	358±40	0.4111
LVDd (mm)	44.5±1.6	44.2±0.50	0.8403
LVDs (mm)	29.8±1.9	27.8±0.63	0.3145
IVS (mm)	10.3±0.60	11.1±0.19	0.1605
PW (mm)	10.3±0.40	10.6±0.14	0.4965
LVEF (%)	56.7±3.5	63.1±1.2	0.0799
LVMI (g/m^2^)	118±8.9	119±3.0	0.9402
E (cm/s)	78.3±9.4	81.6±2.8	0.7373
A (cm/s)	101.9±10.1	106.7±3.0	0.6495
E/A	0.80±0.19	0.80±0.05	0.9901
Dct (ms)	275±28	269±9	0.8246
Septal e' (cm/s)	3.78±0.39	3.86±0.12	0.8502
Lateral e' (cm/s)	6.07±0.51	5.40±0.17	0.2186
Averaged e' (cm/s)	5.22±1.78	4.65±1.29	0.1441
E/e'	16.2±2.6	17.8±0.75	0.5588
Peak AV velocity (m/s)	4.57±0.21	4.53±0.07	0.8488
AV mean PG (mmHg)	50.1±5.0	49.9±1.7	0.9765
AVA (cm^2^)	0.57±0.05	0.66±0.02	0.1107
AR (≧3) (%)	0 (0)	16(13.9)	0.3687
MR (≧3) (%)	3 (23.1)	8 (7.0)	0.084
TR (≧3) (%)	2 (15.4)	5 (4.4)	0.1495
GLS (%)	-11.6±1.2	-15.1±0.4	0.0061
SR_E (/s)	0.71±0.10	0.78±0.03	0.5043
E/SR_E (cm)	142±22.5	122±6.6	0.3801

CAD, coronary artery disease; HT, hypertension; CKD, chronic kidney disease; DM, diabetes mellitus; BNP, brain natriuretic peptide; LVDd, left ventricular diastolic dimension; LVDs, left ventricular systolic dimension; IVS, Interventricular septum; PW, posterior wall; LVEF, left ventricular ejection fraction; LVMI, left ventricular mass index; Dct, deceleration time; AV, aortic valve; peak V, peak velocity; mean PG, mean pressure gradient; AVA, aortic valve area; AR, aortic regurgitation; MR, Mitral regurgitation; TR, tricuspid regurgitation; GLS, global longitudinal strain; SR_E, early diastolic peak strain rate.

**Table 4:** Univariate analysis of echocardiographic parameters for predicting event after TAVI.

	OR	95% CI	P value
LVDd (mm)	1.01	0.91–1.12	0.84
LVDs (mm)	1.04	0.96–1.12	0.32
IVS (mm)	0.76	0.53–1.10	0.15
PW (mm)	0.87	0.59–1.29	0.49
LVEF (%)	0.97	0.93–1.01	0.09
LVMI (g/m^2^)	1.00	0.98–1.02	0.93
E (cm/s)	1.00	0.97–1.02	0.74
A (cm/s)	0.99	0.97–1.02	0.65
E/A	0.99	0.16–6.11	0.99
Dct (ms)	1.00	0.99–1.01	0.83
Averaged e' (cm/s)	1.37	0.90–2.10	0.15
E/e'	0.97	0.88–1.07	0.56
Peak AV velocity (m/s)	1.08	0.50–2.34	0.85
AV mean PG (mmHg)	1.00	0.97–1.03	0.98
AVA (cm^2^)	0.06	0.002–1.97	0.11
GLS (%)	1.23	1.05–1.45	0.004
SR_E (/s)	0.55	0.09–3.19	0.50
E/SR_E (cm)	1.00	1.00–1.01	0.38

LVDd, left ventricular diastolic dimension; LVDs, left ventricular systolic dimension; IVS, Interventricular septum; PW, posterior wall; LVEF, left ventricular ejection fraction; LVMI, left ventricular mass index; Dct, deceleration time; AV, aortic valve; peak V, peak velocity; mean PG, mean pressure gradient; AVA, aortic valve area; GLS, global longitudinal strain; SR_E, early diastolic peak strain rate.

**Fig 2:**
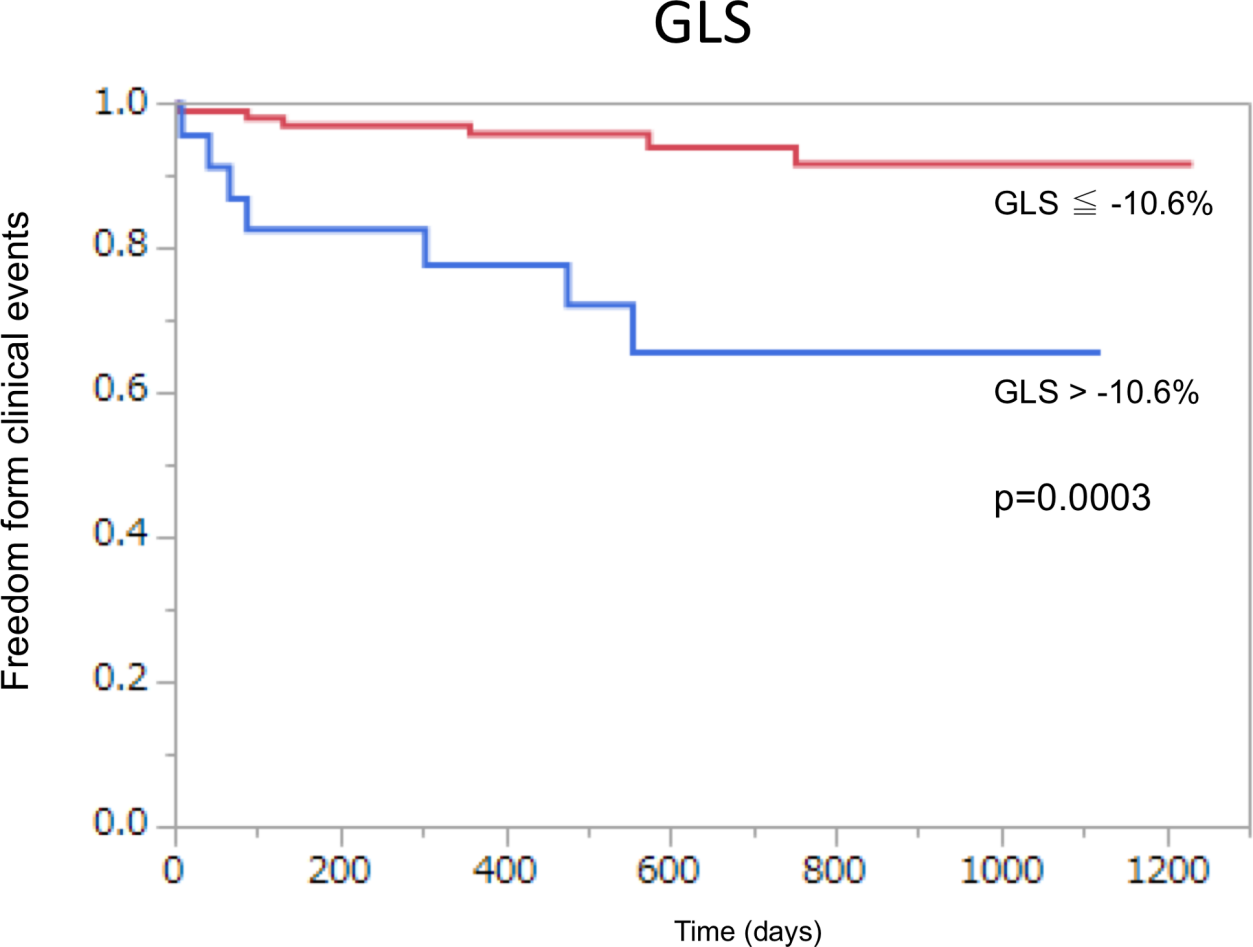
Kaplan-Meier curves showing freedom from event after transcatheter aortic valve implantation. Event-free survival of patients with global longitudinal strain GLS < -10.6% compared to patients with GLS > -10.6%.

Kaplan-Meier analysis revealed that freedom from events for patients with GLS ≦ -10.6% occurred more often than it did for those with GLS > -10.6% (log-rank *P* = 0.0003; Fig 2).

## Discussion

The present study revealed that in patients with severe AS, TAVI improved LV diastolic function (eʹ and SR_E) and LV systolic function (GLS) within 1 week of intervention, and that pre-procedural GLS is a useful predictor for clinical events following TAVI.

Improvements in LV diastolic function have been demonstrated to occur early after TAVI [18, 19]. Consistent with this study, several reports showed that there was an acute improvement in myocardial longitudinal systolic function measured by 2D strain analysis [18, 20, 21]. However, to our knowledge, the present study is the first to demonstrate that pre-procedural GLS could predict the occurrence of clinical events after TAVI.

### Improvement in LV function after TAVI

Wang et al. reported that SR_E was negatively correlated with the time constant of LV pressure decay and that it was stronger than Eʹ [15]. In this study, Eʹ and SR_E were significantly improved after TAVI, indicating that LV diastolic relaxation was ameliorated after TAVI, probably due to the reduction of the LV pressure overload. In contrast, E/SR_E and E/Eʹ, the parameters that correlated with LV filling pressure, unexpectedly worsened due to the increase in E wave velocity. Goncalves et al. previously reported that there was an increase in the E wave maximum velocity immediately after TAVI, and they speculated that this may be explained by alterations in pre-load or LV relaxation [19]. Other studies also reported that E/Eʹ as well as E/SR_E were not improved and were comparable to others, and that these parameters might not reflect the LV filling pressure immediately after TAVI.

In this study, LV systolic function assessed by GLS also improved early after TAVI (within 1 week), although, there was no significant change in LVEF. Consistent with our data, Schastian et al. [21] reported that the radial strain, circumferential strain, and LVEF did not change significantly in all patients immediately after TAVI, but that there was an acute improvement in myocardial longitudinal systolic function measured by 2D strain analysis. They also speculated that GLS could reliably detect early regional changes of myocardial function after TAVI before benefits in LVEF were detectable [21]. This study suggested that not only diastolic function but also systolic function improved within 1 week after TAVI.

### Predictors of clinical outcomes after TAVI

Numerous previous studies have assessed the value of myocardial deformation in predicting clinical outcomes of patients with severe AS [22, 23]. In this study, a worse pre-procedural GLS was significantly associated with clinical events after TAVI, implying that potential LV systolic dysfunction due to LV geometric deformation before TAVI may influence the prognosis after TAVI. LV fibrosis was an independent predictor of mortality for patients with severe aortic stenosis [24–27]. Furthermore, worse GLS was also associated with interstitial fibrosis in an animal model of hypertensive heart failure [28], thereby suggesting that pre-procedural GLS could be a predictor of clinical events after TAVI.

Previous reports have shown that the pre-operative E/SR_E ratio was significantly associated with long-term post-operative survival in patients undergoing SAVR, although the E/SR_E ratio was not significantly different in patients with or without events in this study. This discrepancy might be explained by the different end-points of the two studies (mortality vs. events), since the mortality after TAVI was extremely low in our study. Further studies are needed for clarification.

### Study limitations

There were several limitations to our study. First, this study included only a small number of patients with events. Therefore, multivariate analysis was not conducted. Second, there was no control group of matched patients with conventional SAVR, which limited our ability to assess the efficacy of TAVI.

### Conclusion

Preoperative LV geometric deformation and function, as a consequence of cumulative burden of pressure overload, could predict clinical outcomes after TAVI.
